# Comparative Analysis of Plastome Sequences of Seven *Tulipa* L. (Liliaceae Juss.) Species from Section *Kolpakowskianae* Raamsd. Ex Zonn and Veldk.

**DOI:** 10.3390/ijms25147874

**Published:** 2024-07-18

**Authors:** Shyryn Almerekova, Moldir Yermagambetova, Anna Ivaschenko, Yerlan Turuspekov, Saule Abugalieva

**Affiliations:** 1 Institute of Plant Biology and Biotechnology, Almaty 050040, Kazakhstan; almerekovakz@gmail.com (S.A.); ermaganbetova.moldir@bk.ru (M.Y.); yerlant@yahoo.com (Y.T.); 2 Faculty of Biology and Biotechnology, Al-Farabi Kazakh National University, Almaty 050040, Kazakhstan; 3 Institute of Zoology, Almaty 050040, Kazakhstan

**Keywords:** Liliaceae, *Tulipa*, plastid genome, variable regions, phylogenetic relationships, next-generation sequencing

## Abstract

*Tulipa* L. is a genus of significant economic, environmental, and cultural importance in several parts of the world. The exact number of species in the genus remains uncertain due to inherent taxonomic challenges. We utilized next-generation sequencing technology to sequence and assemble the plastid genomes of seven *Tulipa* species collected in Kazakhstan and conducted a comparative analysis. The total number of annotated genes was 136 in all seven studied *Tulipa* species, 114 of which were unique, including 80 protein-coding, 30 tRNA, and 4 rRNA genes. Nine regions (*petD*, *ndhH*, *ycf2-ycf3*, *ndhA*, *rpl16*, *clpP*, *ndhD-ndhF*, *rpoC2*, and *ycf1*) demonstrated significant nucleotide variability, suggesting their potential as molecular markers. A total of 1388 SSRs were identified in the seven *Tulipa* plastomes, with mononucleotide repeats being the most abundant (60.09%), followed by dinucleotide (34.44%), tetranucleotide (3.90%), trinucleotide (1.08%), pentanucleotide (0.22%), and hexanucleotide (0.29%). The Ka/Ks values of the protein-coding genes ranged from 0 to 3.9286, with the majority showing values <1. Phylogenetic analysis based on a complete plastid genome and protein-coding gene sequences divided the species into three major clades corresponding to their subgenera. The results obtained in this study may contribute to understanding the phylogenetic relationships and molecular taxonomy of *Tulipa* species.

## 1. Introduction

*Tulipa* L. belongs to the Liliaceae Juss. family, and it is a genus with high economic, environmental, and cultural value in many regions of the world [1,2]. The *Tulipa* species are widely distributed in the Middle East, Southern Europe, North Africa, and Central Asia territories [3]. The Tien Shan and Pamir-Alay mountains are the key centers for the diversity of *Tulipa* species in Central Asia [3,4,5]. According to different studies in the literature, the number of species in the genus *Tulipa* varies from 40 to 150 [6,7,8,9,10]. In Kazakhstan, the genus is represented by 42 species and demonstrates adaptability to a wide range of ecological conditions across the country [2]. This adaptability allows *Tulipa* species to thrive in diverse ecosystems, including Kazakhstan’s steppe, meadow, desert, and forest regions [2]. The clarity regarding the number of species and the taxonomy of the genus remains elusive due to a high level of morphological variation in *Tulipa* plants, ongoing discoveries of new species, and the presence of synonyms [9,11]. In addition to the high level of variation between species, a high intra-population genetic diversity level is often reported for a number of studied *Tulipa* species [12]. These studies have typically been conducted using various polymorphic types of DNA markers [13,14,15,16,17,18,19,20,21,22]. Specifically, molecular markers such as random amplified polymorphic DNA (RAPD) [13], inter-simple sequence repeats (ISSRs) [14,15,16], amplified fragment length polymorphisms (AFLPs) [17,18,19], conserved DNA-derived polymorphisms (CDDPs) [20], simple sequence repeats (SSRs) [12], and single-nucleotide polymorphisms (SNPs) [21,22] have been extensively employed to assess the genetic diversity of *Tulipa* species populations. In Kazakhstan, representatives of *Tulipa* also have been studied using botanical methods [23,24]. Quantitative indicators of teratological variability in 26 wild *Tulipa* species from Kazakhstan, both in nature and in cultivation, were analyzed. These indicators can provide valuable insights for future breeding studies [25]. The analysis of the ontogenetic structure of cenopopulations, as well as the morphological and anatomical structure of *T. ostrowskiana* Regel in the Ili Alatau mountains, revealed the influence of the insolation regime of slopes on the age structure and morphometric parameters [26].

Tulipa species have been classified using nuclear genome sizes [1,8] and a phylogenetic study of five plastid regions and the *ITS* region [9]. This classification divides the species into four subgenera, *Clusianae* (Baker) Zonn. and Veldkamp, *Orithyia* (D. Don) Baker, *Eriostemones* (Boiss.) Raamsd., and *Tulipa*, and twelve sections [8]. In Kazakhstan, *Tulipa* species belong to three of the four subgenera mentioned earlier: *Orithyia*, *Eriostemones*, and *Tulipa*. Further, a series of molecular phylogenetic investigations on *Tulipa* species utilizing Sanger sequencing technology have been conducted [27,28,29,30]. Kuhara and coauthors [27] carried out phylogenetic analysis of wild and garden *Tulipa* samples using the coding regions *trnL* and *matK* and the intergenic spacer region *trnT-L* of the plastid genome. Turktas et al. [28] and Hajdari et al. [30] conducted phylogenetic studies using plastid *trnL-trnF* and nuclear ribosomal *ITS* regions to examine the phylogenetic relationships of *Tulipa* species in Turkey and Kosovo, respectively. In a study by Ma et al. [29], DNA barcoding markers (*matK*, *psbA-trnH*, and *rbcL*) were used for species identification of the medicinal plant *T. edulis. matK* was optimal for the identification of *T. edulis* and its adulterants in contrast to *rbcL* and *psbA-trnH* [29]. Recently, an investigation into the phylogenetic relationships within the genus *Tulipa* was conducted using DNA sequences from the *ITS* region [31]. The authors [31] proposed a taxonomic concept consisting of four subgenera (*Tulipa*, *Eriostemones, Orithyia*, and *Clusianae*) and two sections. However, despite these recent attempts to establish a cohesive classification for the genus *Tulipa*, inherent taxonomic challenges persist, suggesting additional studies, including those based on plastid genomes.

The chloroplast, a cellular organelle responsible for photosynthesis, possesses its own genome and is uniparentally inherited in most angiosperms [32]. The structure of the angiosperm plastid genome is typically circular and consists of two inverted repeats (IRs) flanked by a large single-copy (LSC) region and a small single-copy (SSC) region [33,34]. Plastid genome sequences have proven to be efficient tools for assessing phylogenetic relationships [35,36,37], species identification [29], and the identification of polymorphic regions crucial for the development of novel DNA barcode markers [38,39]. 

The general characterization of the plastid genomes of *T. altaica* [40], *T. buhseana* [41], *T. iliensis* [42], *T. patens* [43], *T. gesneriana* [44], and *T. sinkiangensis* [45] have been published and the sequences are available at the National Center for Biotechnology Information (NCBI) database. A comparative analysis of five *Tulipa* plastid genomes was conducted by Li and coauthors [46]. The authors [46] reported that six noncoding regions (*rps16-trnQ*, *trnE-trnT*, *accD-psaI*, *rpl32-trnL*, *rps15-ycf1*, and *rps4-trnT*) and two coding regions (*trnT* and *ycf1*) were highly variable in the studied *Tulipa* plastid genomes. A comparative analysis of the plastid genome in *Tulipa* species growing in Kazakhstan has not been reported in the existing scientific literature. Hence, evaluating genetic variations in the sequences of plastid genomes of the *Tulipa* species from Kazakhstan may provide a wealth of information for the genetic analyses of the genus, including studies related to taxonomic assessments.

The purpose of this study was to analyze the plastid genome sequences of seven *Tulipa* species belonging to the section *Kolpakowskianae* Raamsd. ex Zonn and Veldk. [1], including *T. behmiana* Regel, *T. brachystemon* Regel, *T. kolpakowskiana* Baker, *T. lemmersii* Zonn., A. Peterse and J. de Groot, *T. ostrowskiana* Regel, *T. tetraphylla* Baker, and *T. zenaidae* Vved., collected in Kazakhstan. Almost all studied species are endemic to the territory of Kazakhstan, except for *T. kolpakowskiana*, which is endemic to northern Tien Shan [47], and *T. tetraphylla*, which is native to Central Asia and northwest Xinjiang [48]. The species *T. brachystemon*, *T. kolpakowskiana*, *T. ostrowskiana*, and *T. zenaidae* are listed in the Red Book of Kazakhstan [47].

## 2. Results

### 2.1. Features of the Seven Tulipa Plastomes

**Figure 1:**
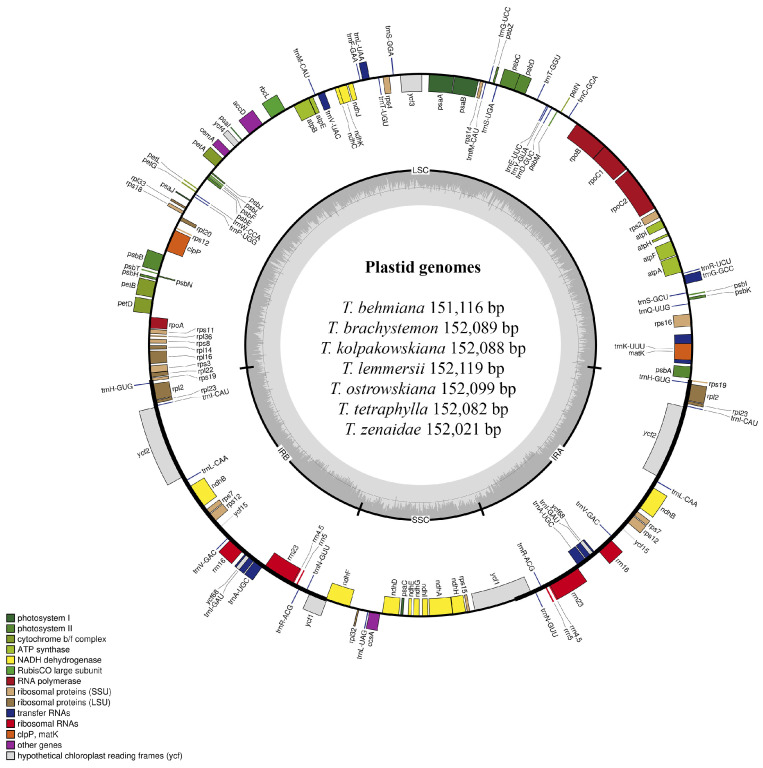
Plastid genome map of *T. behmiana*, *T. brachystemon*, *T. kolpakowskiana*, *T. lemmersii*, *T. ostrowskiana*, *T. tetraphylla*, and *T. zenaidae* species from Kazakhstan. Within the circle, darker grey shades indicate GC content, whereas lighter grey shades indicate AT content. The plastid genome boundaries are divided into the LSC, SSC, IRA, and IRB regions. Genes from various functional groups are color-coded.

Plastid genomes of *T. behmiana*, *T. brachystemon*, *T. kolpakowskiana*, *T. lemmersii*, *T. ostrowskiana*, *T. tetraphylla*, and *T. zenaidae* were sequenced on an Illumina NovaSeq 6000 platform (Illumina Inc., San Diego, CA, USA). The filtered data for all seven species resulted in a final yield exceeding 26 GB. The Q20 quality values were 99.02% for *T. behmiana*, 99.02% for *T. brachystemon*, 98.31% for *T. kolpakowskiana*, 98.15% for *T. lemmersii*, 99.05% for *T. ostrowskiana*, 99.05% for *T. tetraphylla*, and 98.75% for *T. zenaidae.* The Q30 values for *T. behmiana*, *T. brachystemon*, *T. kolpakowskiana*, *T. lemmersii*, *T. ostrowskiana*, *T. tetraphylla*, and *T. zenaidae* were 95.77%, 95.72%, 93.71%, 93.31%, 95.89%, 95.88%, and 94.97%, respectively. The *Tulipa* species’ sequenced plastomes had a typical quadripartite structure (Figure 1), with two copies of inverted repeat regions and two single-copy regions (LSC and SSC).

The total length of the seven *Tulipa* species plastomes ranged in size from 151,116 bp in *T. behmiana* to 152,119 bp in *T. lemmersii*. The LSC region ranged from 81,328 bp (*T. behmiana*) to 82,317 bp (*T. lemmersii*), the SSC region from 17,092 bp (*T. behmiana*) to 17,163 bp (*T. ostrowskiana*), and the IR regions from 56,652 bp (*T. brachystemon*, *T. kolpakowskiana*, and *T. ostrowskiana*) to 56,696 bp (*T. behmiana*). The overall GC content of the assembled plastomes of *T. behmiana*, *T. brachystemon*, *T. kolpakowskiana*, *T. lemmersii*, *T. ostrowskiana*, *T. tetraphylla*, and *T. zenaidae* was 36.69%, 36.65%, 36.65%, 36.63%, 36.64%, 36.63%, and 36.64%, respectively. Whereas the GC content of the LSC regions ranged from 34.53% (*T. tetraphylla*) to 34.62% (*T. behmiana*), that of the SSC regions ranged from 30.08% (*T. behmiana*) to 30.11% (*T. kolpakowskiana* and *T. brachystemon*), and that of the IR regions ranged from 42.02% in *T. behmiana* to 42.05% in *T. brachystemon*, *T. kolpakowskiana*, and *T. ostrowskiana*. The annotated plastid genomes of the seven *Tulipa* species have been deposited in GenBank. All seven analyzed plastid genomes displayed similar gene content and arrangement. The total number of annotated genes was 136 in all seven studied *Tulipa* species, 114 of which were unique, including 80 protein-coding genes, 30 tRNA genes, and 4 rRNA genes (Table 1).

Out of the 136 genes, 22 were duplicated, including10 protein-coding genes (*rps7*, *rps19*, *rps12*, *rpl2*, *rpl23*, *ndhB*, *ycf1*, *ycf2*, *ycf15*, and *ycf68*), 8 tRNA genes (*trnA-UGC*, *trnH-GUG*, *trnI-CAU*, *trnI-GAU*, *trnL-CAA*, *trnN-GUU*, *trnR-ACG*, and *trnV-GAC*) and 4 rRNA genes (*rrn4.5*, *rrn5*, *rrn16*, and *rrn23*). Ten protein-coding genes (*rpoC*, *rps12*, *rps16*, *rpl2*, *rpl16*, *ndhA*, *ndhB*, *petB*, *petD*, and *atpF*) and six tRNA genes (*trnA-UGC*, *trnG-GCC*, *trnI-GAU*, *trnK-UUU*, *trnL-UAA*, and *trnV-UAC*) contained one intron, while two protein-coding genes (*clpP* and *ycf3*) contained two introns. The duplicated copies of the *rps19* and *ycf1* genes and the two copies of the *ycf68* gene found in all seven *Tulipa* plastomes were annotated as pseudogenes (Table 2).

### 2.2. Repeat Sequence Analysis

A total of 1388 simple sequence repeats (SSRs) were identified in the seven *Tulipa* species plastomes by the MIcroSAtellite Identification Tool (MISA). The number of SSRs in the *Tulipa* plastomes ranged from 195 (*T. behmiana*) to 202 (*T. zenaidae*). Among all the identified SSRs, the mononucleotide repeat was the most abundant, comprising 60.09% of the total SSRs, followed by dinucleotide (34.44%), tetranucleotide (3.90%), trinucleotide (1.08%), pentanucleotide (0.22%), and hexanucleotide (0.29%) repeats. The A/T represented a larger proportion of the mononucleotide repeats (800) than C/G repeats (34). The AT/AT content (327) was more abundant than the AG/CT content (151) in dinucleotide repeats. Most of the tetranucleotide repeats consisted of AAAT/ATTT content (27). Pentanucleotide (3) and hexanucleotide (4) repeats were found to be very rare among the seven *Tulipa* plastomes. The pentanucleotide repeat was detected only in *T. ostrowskiana*, *T. tetraphylla*, and *T. zenaidae*, while the hexanucleotide repeat was identified in the plastid genomes of *T. behmiana* and *T. ostrowskiana* (Table 3). Most of the identified SSRs were detected in the intergenic regions of the studied *Tulipa* plastomes (File S1).

**Figure 2:**
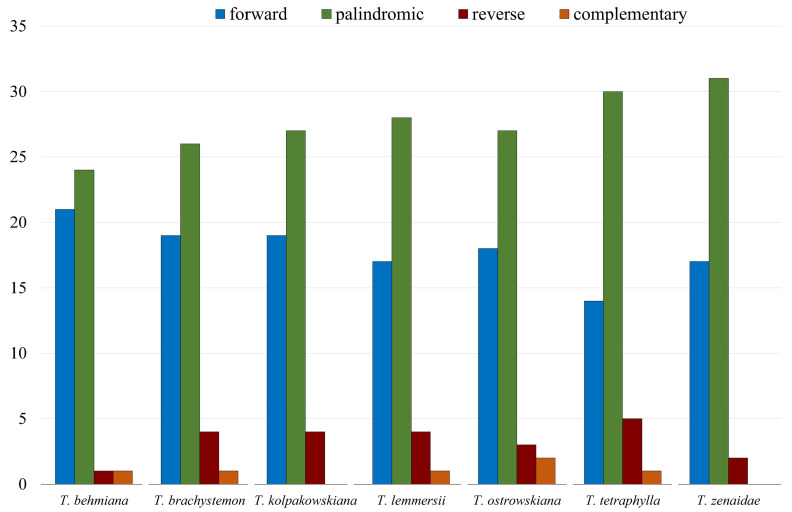
Number of forward, palindromic, reverse, and complementary repeats identified in plastid genomes of *T. behmiana*, *T. brachystemon*, *T. kolpakowskiana*, *T. lemmersii*, *T. ostrowskiana*, *T. tetraphylla*, and *T. zenaidae*.

Other long repeat types, including forward, palindromic, complement, and reverse repeats in the studied *Tulipa* plastomes, were identified. The analysis of the seven *Tulipa* plastomes using REPuter detected 347 repeats. Palindromic repeats (193) were the most abundant type, followed by forward (125), reverse (23), and complementary (6) repeats. Complementary repeats were rare and found only in *T. behmiana* (1), *T. brachystemon* (1), *T. lemmersii* (1), *T. ostrowskiana* (2), and *T. tetraphylla* (1) plastomes. Tandem Repeats Finder recognized 282 tandem repeats in the seven *Tulipa* plastomes, ranging from 37 in *T. zenaidae* to 43 in *T. brachystemon* (Figure 2).

**Figure 3:**
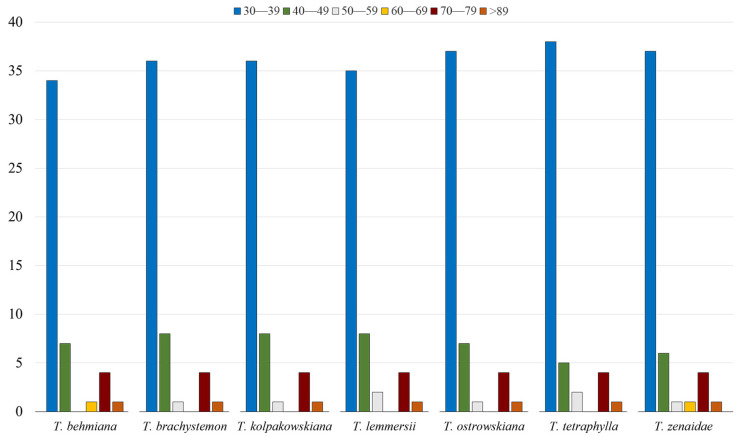
The categorization of long repeats by their lengths in plastid genomes of *T. behmiana*, *T. brachystemon*, *T. kolpakowskiana*, *T. lemmersii*, *T. ostrowskiana*, *T. tetraphylla*, and *T. zenaidae*.

Repeats ranging from 30 to 39 bp were the most abundant in all seven studied plastid genomes, with counts of 34, 36, 36, 35, 37, 38, and 37 for *T. behmiana*, *T. brachystemon*, *T. kolpakowskiana*, *T. lemmersii*, *T. ostrowskiana*, *T. tetraphylla*, and *T. zenaidae*, respectively. Each *Tulipa* plastome contained a single repeat with a length of 89 base pairs or more (Figure 3).

### 2.3. Nucleotide Diversity Analysis

**Figure 4:**
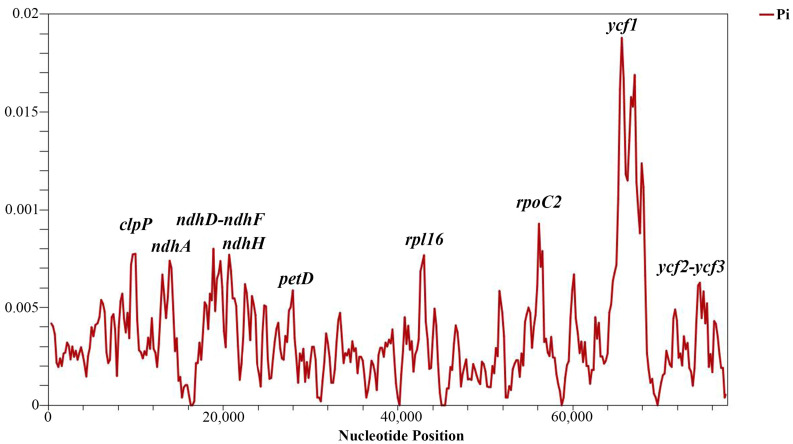
Nucleotide variability (Pi) analysis in 80 protein-coding genes of *Tulipa* plastid genomes using sliding window analysis (window length 600 bp and step size 200 bp). The vertical axis indicates the nucleotide diversity for each window, and the horizontal axis represents the midpoint position.

Sliding window analysis identified highly variable regions in the analyzed protein-coding genes of the *Tulipa* species. The nucleotide variability (Pi) values for the 80 protein-coding genes ranged from 0 to 0.01880, with a mean of 0.00347. Nine regions exhibited relatively high Pi values: *petD*, *ndhH*, *ycf2*-*ycf3*, *ndhA*, *rpl16*, *clpP*, *ndhD*-*ndhF*, *rpoC2*, and *ycf1*. The most variable region was *ycf1*, with a Pi value of 0.02089, indicating more than one variable hotspot (Figure 4). The length, parsimony informative sites, and nucleotide diversity of the variable regions are provided in Table 4.

### 2.4. Selective Pressure Analysis

**Figure 5:**
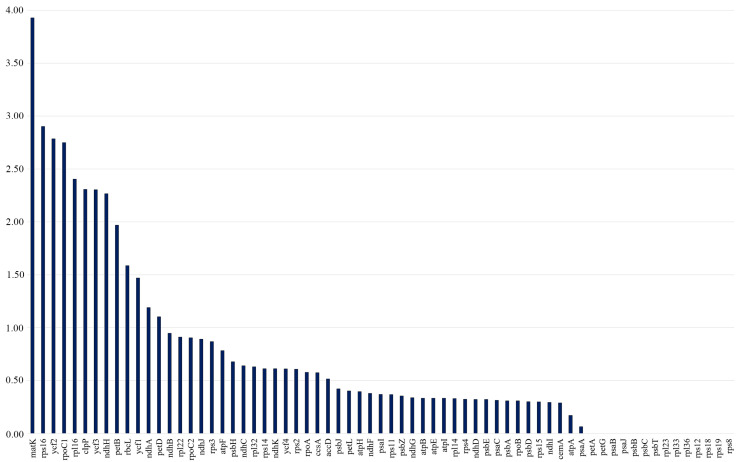
The Ka/Ks ratios of protein-coding genes from 17 *Tulipa* plastid genomes. The vertical axis indicates the Ka/Ks values (ratios), and the horizontal axis represents the protein-coding genes of the plastid genomes.

The Ka/Ks ratios were calculated separately for all 80 protein-coding genes across 17 *Tulipa* plastid genomes. The Ka/Ks values of the protein-coding genes ranged from 0 to 3.9286, with the majority of the genes having Ka/Ks values less than 1. It was indicated that matK had the highest average Ka/Ks ratio of 3.9286, followed by *rps16* (2.9024), *ycf2* (2.7857), *rpoC1* (2.7500), *rpl16* (2.4048), *clpP* (2.3077), *ycf3* (2.3043), *ndhH* (2.2667), *petB* (1.9688), *rbcL* (1.5862), *ycf1* (1.4694), *ndhA* (1.1905), and *petD* (1.1039), suggesting positive selection. The Ka/Ks values of the remaining 54 protein-coding genes were all less than 1, indicating that these genes were under purifying selection (Figure 5).

### 2.5. Contraction and Expansion of the Inverted Repeat Regions

**Figure 6:**
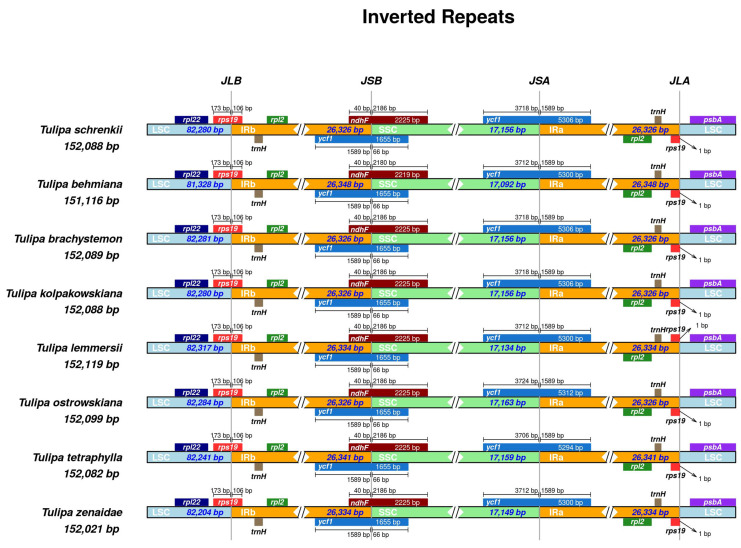
Comparisons of the borders of the LSC, IR, and SSC regions among *Tulipa* plastomes. JLB indicates the junction sites between the LSC and IRb regions, JSB indicates the junction sites between the IRb and SSC regions, JSA indicates the junction sites between the SSC and IRa regions, and JLA indicates the junction sites between the IRa and LSC regions.

Analysis of contraction and expansion in the junction regions of LSC, SSC, and IR was conducted in the seven studied *Tulipa* plastomes with *T. schrenkii* (NC063594.1) as the reference. In all compared *Tulipa* plastomes, the placement of the *rpl22* and *psbA* genes was exclusively within the LSC region. Conversely, both the *rpl2* and *trnH* genes were located entirely in the IR regions. The positioning of the *rps19* genes occurred at the IRa/LSC and LSC/IRb junctions, with the integration of 106 bp into the *IRb*. The genes *ndhF* and *ycf1* extended beyond the IRb/SSC borders, incorporating 40 bp and 1589 bp into the IRb region. Furthermore, a duplicate copy of the *ycf1* gene was evident at the IRa/SSC junction in all *Tulipa* plastomes, integrated into the IRa by 1589 bp (Figure 6).

### 2.6. Phylogenetic Analysis

**Figure 7:**
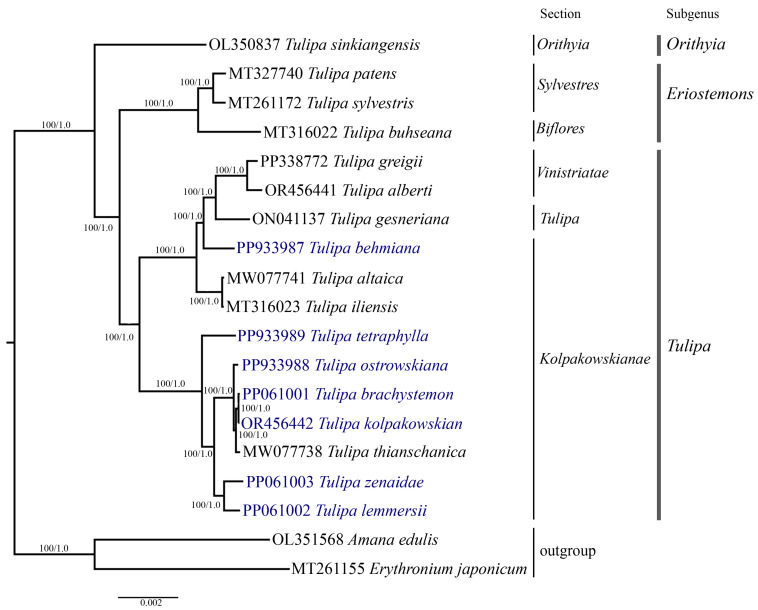
The phylogenetic tree was reconstructed using complete plastid genome sequences from 17 *Tulipa* species and two outgroup species, employing both Maximum Likelihood (ML) and Bayesian Inference (BI) methods. The numbers at the branch nodes represent ML bootstrap/BI posterior probability values. The species analyzed in this study are highlighted in blue.

**Figure 8:**
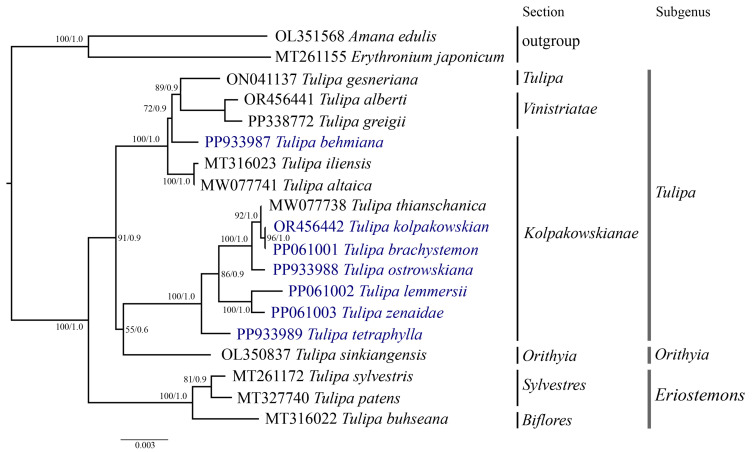
Phylogenetic tree inferred from *ycf1* gene nucleotide sequences for 17 *Tulipa* species and two outgroup species using Maximum Likelihood (ML) and Bayesian Inference (BI) methods. The numbers at the branch nodes represent ML bootstrap/BI posterior probability values. The species analyzed in this study are highlighted in blue.

Phylogenetic analysis was conducted based on the nucleotide sequences of complete plastid genomes (Figure 7), protein-coding genes (File S2), and the *ycf1* (Figure 8) gene of 19 samples, including the seven *Tulipa* species analyzed in this study, ten Tulipa species from GenBank, and two outgroup samples (*Amana edulis* and *Erythronium japonicum*) using the Maximum Likelihood (ML) and Bayesian inference (BI) methods. The phylogenetic trees derived from the nucleotide sequences of the complete plastid genome (Figure 7) and protein-coding genes (File S2) exhibited similar topologies, clustering the *Tulipa* species into three major clades corresponding to the subgenera *Eriostemons*, *Tulipa*, and *Orithyia*. The phylogenetic tree based on the *ycf1* gene (Figure 8) nucleotide sequences grouped the species into two major clades, with species from the subgenus *Orithyia* being mixed together with species from the subgenus *Tulipa*.

Six *Tulipa* species collected in Kazakhstan (*T. brachystemon*, *T. kolpakowskiana*, *T. lemmersii*, *T. ostrowskiana*, *T. tetraphylla*, and *T. zenaidae*) formed one subclade with *T. thianschanica* from GenBank, all belonging to the section *Kolpakowskianae* of the subgenus Tulipa. The seventh species (*T. behmiana*) from Kazakhstan from the same section formed a separate subclade with species from the sections *Tulipa* and *Vinistriatae*, also within the subgenus *Tulipa*.

## 3. Discussion

In the present study, we have assembled the plastome sequences of seven *Tulipa* (*T. behmiana*, *T. brachystemon*, *T. kolpakowskiana*, *T. lemmersii*, *T. ostrowskiana*, *T. tetraphylla*, and *T. zenaidae*) species from Kazakhstan and conducted a comparative analysis. The plastid genome of seven *Tulipa* species has a typical quadripartite structure consisting of two IR regions separated by the LSC and SSC regions. A greater proportion of GC content more than 40% was identified within the IR (IRa and IRb) region in contrast to the LSC and SSC regions. This observation aligns consistently with data reported in previous studies on *Tulipa* species [46]. The increased GC content identified in the IR region may be a consequence of the presence of four rRNA (*rrn4.5*, *rrn5*, *rrn16*, and *rrn23*) genes [49,50].

The comparative analysis of the seven studied *Tulipa* genomes revealed 114 unique genes, including 80 protein-coding genes, 30 tRNA genes, and 4 rRNA genes. Plastid genomes often exhibit variation in gene content, and pseudogenes are identified as non-functional remnants of once-active genes [51]. We annotated four pseudogenes (duplicated copies of the *rps19* and *ycf1* genes and two copies of the *ycf68* gene) in the studied *Tulipa* plastomes. The pseudogenization of *ycf1*, *ycf68*, the exact function of which remains unclear, and *rps19*, which encodes ribosomal protein S19 in plastid genomes, is well documented [52,53,54], reflecting the dynamic evolution of plant genomes involving processes such as functional transfer to the nuclear genome [55]. The genes *ycf1*, *ycf68*, and *rps19* are common in *Tulipa* plastid genomes and are often annotated as pseudogenes [45,46,56]. Moreover, the structural variations at the junction sites of LSC, SSC, and IR regions, as reported in prior studies [46,57], were partially attributed to the presence of the pseudogenes *ycf1* and *rps19*, which were identified in this study. The loss of the *infA* gene is evident across all seven examined plastid genomes. Comparative analyses with the plastid genomes of various *Tulipa* species, including *T. altaica* [40,46], *T. buhseana* [41], *T. iliensis* [42,46], *T. patens* [43,46], *T. gesneriana* [44], *T. sinkiangensis* [45], *T. thianschanica*, and *T. sylvestris* [40,46], reveals the absence of this gene in these species as well. The loss of the *infA* gene, which codes for translation initiation factor 1, may be attributed to its transfer from the plastid to the nuclear or mitochondrial genomes during angiosperm evolution [58]. The loss of the *infA* gene appears to occur independently and is not a unique phenomenon in the plastid genomes of angiosperms. Persistent exploration in this field holds the potential to reveal a more comprehensive understanding of the functional implications and evolutionary significance associated with pseudogenes or gene loss events.

The Ka/Ks ratio measures the selection pressure on a gene, indicating positive selection when Ka/Ks > 1 and purifying selection when Ka/Ks < 1 [59]. In this study, Ka/Ks analysis revealed that most genes (54) were under purifying selection, while 12 genes (*rps16*, *ycf2*, *rpoC1*, *rpl16*, *clpP*, *ycf3*, *ndhH*, *petB*, *rbcL*, *ycf1*, *ndhA*, and *petD*) were under positive selection. These results confirm that protein-coding genes are typically characterized by purifying selection [37,39].

SSRs, also known as microsatellites, have garnered significant attention in genetic research due to their abundance, polymorphic nature, and wide applicability [60,61,62]. The utilization of SSR markers provides valuable insights into genetic diversity, population structure, and evolutionary processes [63,64,65]. In our investigation, we identified 1388 SSRs across the plastomes of seven *Tulipa* species. Notably, mononucleotide repeats constituted the majority (60.09%) of the detected SSRs, with A/T being the most prevalent nucleotide content. These findings align with and confirm the outcomes reported in previous studies [57,66]. The intergenic regions in the seven *Tulipa* plastomes exhibited the highest percentage of identified SSRs, aligning with similar analyses conducted in other representatives of the Liliaceae family [67,68]. Furthermore, within the studied *Tulipa* plastomes, we identified palindromic, forward, reverse, complementary, and tandem repeats, with palindromic repeats (193) notably more abundant than forward, reverse, complement, and tandem repeats. Likewise, palindromic repeats emerge as the dominant repeat type in various plant species, such as within the genera *Lilium* [69], *Polyspora* [70], and *Cicer* [71]. Repetitive sequences are pivotal in facilitating genome rearrangements and are frequently utilized in phylogenetic studies to elucidate evolutionary relationships [72,73].

The polymorphic regions within plastid genomes can serve as molecular markers, offering valuable data for DNA barcoding and phylogenetic analysis [74,75,76]. In this study, the region of the *ycf1* gene nucleotide sequences displayed the highest degree of polymorphism and was used for the phylogenetic analysis. The results obtained in this study are consistent with previous findings [57,77] and propose the potential use of these outcomes as DNA barcoding markers within *Tulipa*.

Initially, our focus was on sequencing the nuclear *ITS* region, as previously we successfully sequenced the *ITS* region and assessed the phylogeny of several genera, including *Ranunculus* [78], *Allium* [79], and *Oxytropis* [80]. However, the obtained results suggested the inefficiency of the *ITS* region for categorizing *Tulipa* species into their respective sections or subgenera (unpublished data). The literature survey showed that most of the conducted phylogenetic studies on *Tulipa* species have utilized regions of the plastid genome [27,28,29,30]. In this study, the data obtained from the comparative study of seven new and ten known plastid genome sequences proved to be informative and effective for molecular taxonomy analysis of *Tulipa* species.

Plastid genome sequences have previously been extensively utilized for phylogenetic reconstructions to elucidate the evolutionary relationships among plant species and resolve taxonomic ambiguities [53,54]. *Tulipa* is recognized as a taxonomically controversial genus due to the presence of diverse morphological characteristics within its species [9]. Molecular phylogenetic analysis of *Tulipa* has been carried out on a relatively small number of genetic loci, including plastid and nuclear DNA markers [9,11,27,28,29,30]. In this study, a phylogenetic tree was constructed based on the nucleotide sequences of the complete plastid genome (Figure 7), protein-coding genes (File S2), and *ycf1* (Figure 8) gene. The dataset incorporated samples from 17 distinct *Tulipa* species, including seven species from Kazakhstan and two outgroup species. The species from section *Kolpakowskianae*, including those analyzed in this study (*T. behmiana*, *T. brachystemon*, *T. kolpakowskiana*, *T. lemmersii*, *T. ostrowskiana*, *T. tetraphylla*, and *T. zenaidae*) formed a single large clade along with species from the *Tulipa* and *Vinistriatae* sections in the analysis. These findings support the taxonomic concept proposed by B. Wilson [81] and Eker et al. [31], which merges the sections *Vinistriatae* and *Kolpakowskianae* into a single section named *Tulipa*.

In addition, the results from this study may provide valuable information for future *Tulipa* germplasm conservation strategies. Specifically, we identified over 1300 putative SSRs in the plastid genome sequences of seven *Tulipa* species. These SSRs can serve as potential DNA markers for population genetic studies, helping to identify the most genetically diverse populations and develop effective conservation strategies. Moreover, we identified polymorphic regions in the plastid genomes of the analyzed *Tulipa* species to find potential DNA barcoding markers that can be informative for the taxonomy of the genus. In this study, the *ycf1* gene region was recognized as the most polymorphic, suggesting that the *ycf1* gene can be used as a specific barcode for *Tulipa* species.

## 4. Materials and Methods

### 4.1. Plant Material and DNA Extraction

The plant leaves of seven *Tulipa* species (*T. behmiana*, *T. brachystemon*, *T. kolpakowskiana*, *T. lemmersii*, *T. ostrowskiana*, *T. tetraphylla*, and *T. zenaidae*) were collected from the Almaty, Zhambyl, and Turkestan regions of Kazakhstan. Permission was obtained to collect plant leaves of *Tulipa* species from the Forestry and Wildlife Committee of the Ministry of Ecology, Geology, and Natural Resources of the Republic of Kazakhstan. The collected fresh leaves were dried in silica gel for further DNA extraction. Genomic DNA was extracted, using the cetyltrimethylammonium bromide (CTAB) protocol [82], from the dried leaves of *Tulipa* samples.

### 4.2. Sequencing, Assembly, and Annotation

The sequencing part of the study was carried out according to our previous reports on the characterization of plastid genomes in *Juniperus* species [83,84,85]. DNA samples that successfully passed quality control assessments were used to prepare paired-end libraries with the TruSeq Nano DNA Kit (Illumina Inc., San Diego, CA, USA). Paired-end sequencing of the seven *Tulipa* species from Kazakhstan was conducted on an Illumina NovaSeq 6000 platform (Illumina Inc., San Diego, CA, USA) at Macrogen Inc. (Seoul, Republic of Korea). The quality control of raw reads was checked using FastQC (https://www.bioinformatics.babraham.ac.uk/projects/fastqc/, accessed on 27 May 2024) and trimmed using Trimmomatic 0.36 [86] to remove adapter sequences. Clean reads were assembled using NOVOPlasty version 4.3.3 [87]. The assembled plastome sequences of the seven *Tulipa* species were annotated using GeSeq [88] and PGA [89]. They were then manually corrected by comparing them with *T. schrenkii* (NC063594.1) as the reference. The annotated sequences of *T. behmiana*, *T. brachystemon*, *T. kolpakowskiana*, *T. lemmersii*, *T. ostrowskiana*, *T. tetraphylla*, and *T. zenaidae* plastomes obtained in this study have been deposited into the NCBI database under accession numbers PP933987, PP061001, OR456442, PP061002, PP933988, PP933989, and PP061003, respectively. The circular plastid genome map was drawn by Organellar Genome DRAW 1.3.1 (OGDRAW) [90].

### 4.3. Repeat Element Analysis, Nucleotide Diversity, IR Region Contraction and Expansion, and Ka/Ks Ratio Analysis

Tandem repeat sequences were detected using the Tandem Repeats Finder program [91] with the default settings. The REPuter web-based program [92] was used to find the forward (F), palindromic (P), reverse (R), and complementary (C) repeat elements with the following parameter settings: Hamming distance = 3 and minimum repeat size = 30 bp. The position and types of simple sequence repeats (SSRs) were detected using MISA online tool (https://webblast.ipk-gatersleben.de/misa/, accessed on 19 June 2024) [93] with thresholds for mononucleotide SSRs—eight repeats; for dinucleotide and trinucleotide—four repeats; and for tetranucleotide, pentanucleotide, and hexanucleotide—three repeats. The DnaSP 6 (DNA Sequence Polymorphism) package [94], with a 200 bp step size and a 600 bp window length, was used to calculate nucleotide diversity (Pi) for the aligned protein-coding genes of all *Tulipa* species. We separately isolated and aligned the 80 protein-coding genes to evaluate synonymous (Ks) and nonsynonymous (Ka) substitution rates. Subsequently, each gene’s Ka/Ks ratios were analyzed using DnaSP 6 [94]. The seven plastid genome and reference sample *T. schrenkii* (NC063594.1) junction sites were analyzed and visualized using IRscope (https://irscope.shinyapps.io/irapp/, accessed on 24 June 2024) online [95].

### 4.4. Phylogenetic Analysis

Phylogenetic analysis was conducted based on the alignment of the sequences of the complete plastid genome, protein-coding genes, and the *ycf1* gene of seven *Tulipa* species collected in Kazakhstan, ten *Tulipa* samples from NCBI, and two outgroup samples, *Amana edulis* (OL351568) and *Erythronium japonicum* (MT261155). The program Geneious Prime^®^ 2024.0.2 (https://www.geneious.com) was used for the alignment of nucleotide sequences. Phylogenetic analysis was conducted using the Maximum Likelihood (ML) and Bayesian inference (BI) methods. ML phylogenetic trees were reconstructed based on the TVM + F + I (complete plastid genome sequences), TVM + F + I + R4 (protein-coding genes), and K3Pu + F + G4 (*ycf1* gene) best nucleotide substitution model according to Bayesian information criterion (BIC) using IQ-TREE 2.2.2.6 software [96]. BI trees were constructed using MrBayes 3.2.7 [97] with the following parameters: ngen = 3,000,000, samplefreq = 200, and burninfrac = 0.25. The resulting trees were visualized using FigTree 1.4.4 (http://tree.bio.ed.ac.uk/software/figtree/, accessed on 20 June 2024). The subgenus and section names of the *Tulipa* species in this analysis were given according to Veldkamp and Zonneveld [1].

## 5. Conclusions

In this study, the complete plastid genomes of seven *Tulipa* species (*T. behmiana*, *T. brachystemon*, *T. kolpakowskiana*, *T. lemmersii*, *T. ostrowskiana*, *T. tetraphylla*, and *T. zenaidae*) collected in Kazakhstan were analyzed. The structure and gene content of the studied plastid genomes of the seven *Tulipa* species were similar. The total length of the studied *Tulipa* plastomes ranged in size from 151,116 bp in *T. behmiana* to 152,119 bp in *T. lemmersii*. The total number of annotated genes was 136 in all seven studied *Tulipa* species, 114 of which were unique, including 80 protein-coding, 30 tRNA, and 4 rRNA genes. The analysis of the seven plastid genomes revealed the presence of 1388 simple sequence repeats, with counts ranging from 195 in *T. behmiana* to 202 in *T. zenaidae*. The nucleotide variability (Pi) values for the 80 protein-coding genes ranged from 0 to 0.01880. Among these genes, nine regions showed relatively high Pi values: *petD*, *ndhH*, *ycf2-ycf3*, *ndhA*, *rpl16*, *clpP*, *ndhD-ndhF*, *rpoC2*, and *ycf1*. Notably, *ycf1* exhibited the highest variability (0.02089), indicating the presence of multiple variable hotspots. The *ycf1* gene, along with complete plastid genome and protein-coding gene nucleotide sequences, was used for the phylogenetic analysis. The phylogenetic trees generated using Maximum Likelihood and Bayesian inference methods divided the species into three main clades corresponding to their respective subgenera. The nucleotide sequences of the *ycf1* gene from the plastid genome can be recommended as a potential DNA barcoding marker for the genus *Tulipa*. The obtained nucleotide sequences of the seven *Tulipa* species may prove suitable for phylogenetic analysis and molecular taxonomy.

## Figures and Tables

**Table 1:** Features of plastid genomes in seven *Tulipa* species.

	*T. behmiana*	*T. brachystemon*	*T. kolpakowskiana*	*T. lemmersii*	*T. ostrowskiana*	*T. tetraphylla*	*T. zenaidae*
GenBank numbers	PP933987	PP061001	OR456442	PP061002	PP933988	PP933989	PP061003
Genome size (bp)	151,116	152,089	152,088	152,119	152,099	152,082	152,021
LSC (bp)	81,328	82,281	82,280	82,317	82,284	82,241	82,204
SSC (bp)	17,092	17,156	17,156	17,134	17,163	17,159	17,149
IR (bp)	52,696	52,652	52,652	52,668	52,652	52,682	52,668
Number of total genes	136	136	136	136	136	136	136
Protein-coding genes	80	80	80	80	80	80	80
tRNAs	30	30	30	30	30	30	30
rRNAs	4	4	4	4	4	4	4
Total GC content (%)	36.69	36.65	36.65	36.63	36.64	36.63	36.64
LSC GC content (%)	34.62	34.55	34.56	34.54	34.55	34.53	34.56
SSC GC content (%)	30.08	30.11	30.11	30.09	30.09	30.09	30.10
IR GC content (%)	42.02	42.05	42.05	42.03	42.05	42.04	42.03

**Table 2:** List of genes annotated in seven plastomes of *Tulipa* species.

Category	Group of Genes	Name of Genes
Self-replication	Transfer RNA	*trnA-UGC* ^a^ (×2), *trnC-GCA*, *trnD-GUC*, *trnE-UUC*, *trnF-GAA*, *trnfM-CAU*, *trnG-GCC* ^a^, *trnG-UCC*, *trnH-GUG* (×2), *trnI-CAU* (×2), *trnI-GAU* ^a^ (×2), *trnK-UUU* ^a^, *trnL-CAA* (×2), *trnL-UAA* ^a^, *trnL-UAG*, *trnM-CAU*, *trnN-GUU* (×2), *trnP-UGG*, *trnQ-UUG*, *trnR-ACG* (×2), *trnR-UCU*, *trnS-GCU*, *trnS-GGA*, *trnS-UGA*, *trnT-GGU*, *trnT-UGU*, *trnV-GAC* (×2), *trnV-UAC* ^a^, *trnW-CCA*, *trnY-GUA*
	Ribosomal RNA	*rrn4.5* (×2), *rrn5* (×2), *rrn16* (×2), *rrn23* (×2)
	RNA polymerase	*rpoA*, *rpoB*, *rpoC1* ^a^, *rpoC2*
	Small subunit of ribosome	*rps2*, *rps3*, *rps4*, *rps7* (×2), *rps8*, *rps11*, *rps12* ^a^ (×2), *rps14*, *rps15*, *rps16* ^a^, *rps18*, *rps19*, *rps19* ^c^
	Large subunit of ribosome	*rpl2* ^a^ (×2), *rpl14*, *rpl16* ^a^, *rpl20*, *rpl22*, *rpl23* (×2), *rpl32*, *rpl33*, *rpl36*
Genes for photosynthesis	NADH dehydrogenase	*ndhA* ^a^, *ndhB* ^a^ (×2), *ndhC*, *ndhD*, *ndhE*, *ndhF*, *ndhG*, *ndhH*, *ndhI*, *ndhJ*, *ndhK*
	Photosystem I	*psaA*, *psaB*, *psaC*, *psaI*, *psaJ*
	Photosystem II	*psbA*, *psbB*, *psbC*, *psbD*, *psbE*, *psbF*, *psbH*, *psbI*, *psbJ*, *psbK*, *psbL*, *psbM*, *psbN*, *psbT*, *psbZ*
	Subunits of cytochrome	*petA*, *petB* ^a^, *petD* ^a^, *petG*, *petL*, *petN*
	ATP synthase	*atpA*, *atpB*, *atpE*, *atpF* ^a^, *atpH*, *atpI*
	Rubisco	*rbcL*
Other genes	Maturase	*matK*
	Protease	*clpP* ^b^
	Envelope membrane protein	*cemA*
	Subunit of acetyl-CoA-carboxylase	*accD*
	C-type cytochrome synthesis gene	*ccsA*
Genes of unknown function	Hypothetical chloroplast reading frames	*ycf1*, *ycf1* ^c^, *ycf2* (×2), *ycf3* ^b^, *ycf4*, *ycf15* (×2), *ycf68* ^c^, *ycf68* ^c^

^a^ one-intron-containing gene; ^b^ two-intron-containing gene; ^c^ pseudo gene; (×2) duplicated gene.

**Table 3:** Types and amounts of simple sequence repeat markers (SSRs) in the plastomes of seven *Tulipa* species.

Type	Repeat Unit	*T. behmiana*	*T. brachystemon*	*T. kolpakowskiana*	*T. lemmersii*	*T. ostrowskiana*	*T. tetraphylla*	*T. zenaidae*	Total	%
Mono-	A/T	110	115	115	117	115	118	110	800	60.09
	C/G	4	5	5	5	5	5	5	34	
Di-	AT/AT	44	48	48	45	47	44	51	327	34.44
	AG/CT	22	21	21	22	21	22	22	151	
Tri-	AAT/ATT	4	1	1	2	1	2	4	15	1.08
Tetra-	AAAT/ATTT	4	4	4	3	4	3	5	27	3.89
	AAAG/CTTT	1	1	1	1	1	1	1	7	
	AATT/AATT	1	1	1	1	1	1	1	7	
	AGAT/ATCT	1	1	1	1	1	1	1	7	
	AATG/ATTC	1	1	1	-	1	1	1	6	
Penta-	AAAAG/CTTTT	-	-	-	-	1	-	1	2	0.22
	AATAT/ATATT	-	-	-	-	-	1	-	1	
Hexa-	AAAAAC/GTTTTT	2	-	-	-	-	-	-	2	0.29
	ATCATG/ATCATG	1	-	-	-	-	-	-	1	
	AAACAG/CTGTTT	-	-	-	-	1	-	-	1	
Total		195	198	198	197	199	199	202	1388	

**Table 4:** Highly variable regions in the nucleotide sequences of protein-coding genes among the analyzed *Tulipa* species.

Variable Region	Length	Variable Sites	Parsimony Informative Sites	Nucleotide Diversity
*petD*	650	15	5	0.00588
*ndhH*	600	13	9	0.00620
*ycf2-ycf3*	612	12	7	0.00627
*ndhA*	616	18	8	0.00740
*rpl16*	606	15	8	0.00768
*clpP*	709	22	11	0.00775
*ndhD-ndhF*	600	17	11	0.00801
*rpoC2*	600	25	12	0.00929
*ycf1*	615	36	24	0.01880

## Data Availability

All data are contained within the article and its Supplementary Files.

## Supplementary Materials

The following supporting information can be downloaded at: https://www.mdpi.com/article/10.3390/ijms25147874/s1.
