# Supplementary material for: Comparative Analysis of Plastome Sequences of Seven Tulipa L. (Liliaceae Juss.) Species from Section Kolpakowskianae Raamsd. Ex Zonn and Veldk
Source: Int J Mol Sci. 2024 Jul 18;25(14):7874. doi: 10.3390/ijms25147874 (PMC11277319; doi:10.3390/ijms25147874)
Supplement: Supplementary file 1 [file ijms-25-07874-s001.zip › File S2.pdf]

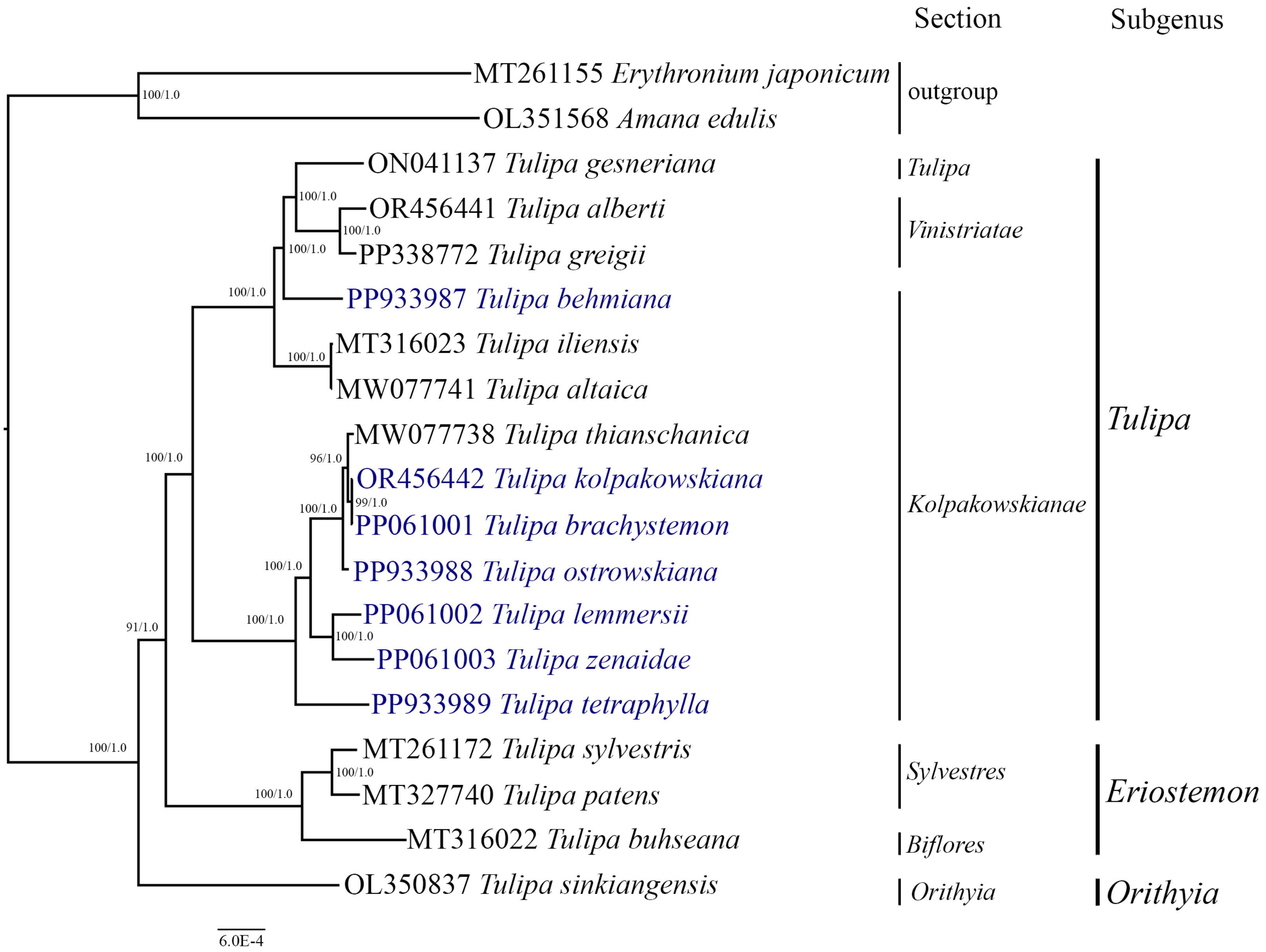

Phylogenetic tree reconstructed using sequences of 80 protein-coding genes from 17 *Tulipa* species and two outgroup species, employing Maximum Likelihood (ML) and Bayesian Inference (BI) methods. The numbers at the branch nodes represent ML bootstrap/BI posterior probability values. The species analyzed in this study are highlighted in blue.
